# Moderate-dose doxorubicin does not affect right ventricular size or function parameters in lymphoma patients—a single-center study

**DOI:** 10.1093/ehjopen/oeag039

**Published:** 2026-03-13

**Authors:** Eduardo Brenner-Muslera, Maria F Gomez-Ardila, Eduardo Tellez-Garcia, James R Cerhan, Hector R Villarraga

**Affiliations:** Division of Cardiovascular Ultrasound, Department of Cardiovascular Diseases, Mayo Clinic, 200 First St SW, Rochester, MN 55905, USA; Division of Cardiovascular Ultrasound, Department of Cardiovascular Diseases, Mayo Clinic, 200 First St SW, Rochester, MN 55905, USA; Division of Cardiovascular Ultrasound, Department of Cardiovascular Diseases, Mayo Clinic, 200 First St SW, Rochester, MN 55905, USA; Division of Epidemiology Department of Quantitative Health Sciences, Mayo Clinic, 200 First St SW, Rochester, MN 55905, USA; Division of Cardiovascular Ultrasound, Department of Cardiovascular Diseases, Mayo Clinic, 200 First St SW, Rochester, MN 55905, USA

**Keywords:** Anthracyclines, Cardiotoxicity, Echocardiography, Right ventricle, Strain

## Abstract

**Aims:**

We sought to determine whether doxorubicin affected echocardiographic parameters of right ventricular (RV) size and function when compared with guideline defined values in patients with lymphoma.

**Methods and results:**

We enrolled 356 patients with lymphoma from 2013 to 2020. Patients received a mean (SD) of 5.12 (1.48) chemotherapy cycles and a cumulative, body surface area–adjusted doxorubicin dosage of 243.6 (79.6) mg/m^2^. Echocardiographic assessments occurred at 3 time points: T0, baseline; T1, 3 to 6 months after starting doxorubicin (mean [SD], 5.1 [1.2] months); and T2, 6 to 18 months (mean [SD], 12.98 [2.6] months) after treatment. End-diastolic area remained unchanged (*P* = 0.12). End-systolic area increased from 10.55 cm^2^ at T0 to 11.41 cm^2^ at T1 and 11.32 cm^2^ at T2 (*P* < 0.001). Fractional area change decreased from 46.67% at T0 to 44.35% at T1 and 44.40% at T2 (P = 0.003). Tricuspid annular plane systolic excursion decreased from 23.17 (4.14) mm to 22.39 (4.10) mm at T1, and 22.13 (3.88) mm at T2 (*P* = 0.006). S′ declined from 0.14 m/s at T0 to 0.13 m/s at T1 and T2 (*P* < 0.001). Tricuspid regurgitant velocity (*P* = 0.58) and RV systolic pressure (*P* = 0.75) showed nonsignificant changes. RV free wall strain worsened from −26.29% at T0 to −25.37% at T2 (*P* = 0.001). All parameters remained within guideline defined values (*P* < 0.001). No significant differences were noted across dosage groups: <200, 200–300, and >300 mg/m^2^.

**Conclusion:**

Echocardiography derived parameters of right ventricular size and function remained within normal guideline-defined values throughout a follow-up of up to 12.98 ± 2.6 months.

## Structured Graphical Abstract

**Structured Graphical Abstract:**
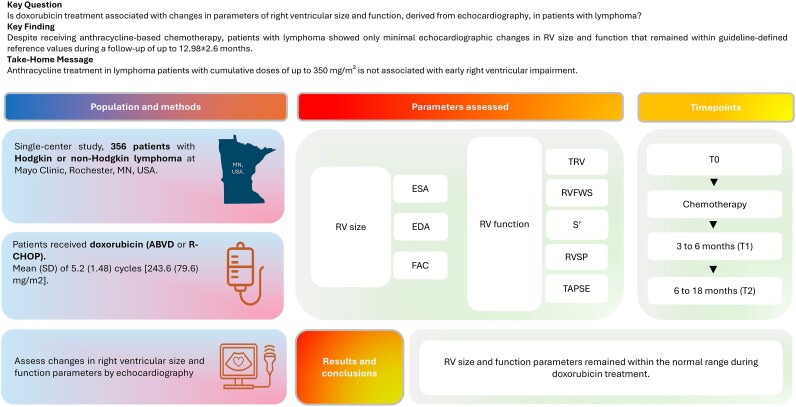

## Introduction

Advancements in cancer screening techniques and treatment during the past three decades have markedly increased survival rates.^1^ Lymphoma is no exception, and many patients have become long-term survivors. However, this growing population faces an increased risk of cardiac complications from treatment- and nontreatment-related causes. Most studies of these complications have focused on early detection of left ventricular (LV) cardiotoxicity, subclinical dysfunction, or overt heart failure.^2^

Anthracyclines (AC), some of the most commonly used chemotherapeutic agents, are known to have cardiotoxic effects; most data supporting this association are derived from long-term breast cancer registries and studies of childhood cancer survivors. Recent evidence shows that any type of AC exposure, regardless of dosage, is variably associated with an increased risk of adverse cardiovascular outcomes, especially heart failure.^3,4^ The St Jude Lifetime Cohort Study examined the prevalence of cardiac dysfunction among adult survivors of childhood cancer, particularly those exposed to AC chemotherapy and chest-directed radiotherapy.^5^ Among 1820 survivors, only 5.8% had reduced LV ejection fraction (EF). However, 32.1% of those with normal LV EF showed cardiac dysfunction, with abnormal LV global longitudinal strain (GLS; 28% of patients) or diastolic dysfunction (8.7%).

Prior studies have shown that LV and right ventricle (RV) cardiotoxicity are not always present at the same time or in the same patient. For instance, Laufer-Perl *et al*.^6^ investigated the effect of AC therapy on LV and RV function in 40 women with breast cancer. Although LV function (as measured by LV EF and LV GLS) remained within normal values, the study showed a significant decrease in both RV GLS and RV free wall strain (RVFWS), with 75% and 58% of patients showing reductions of at least 10% in these parameters, respectively. Therefore, the authors concluded that early, subclinical cardiotoxicity could be identified on the basis of RV changes, even in the absence of LV dysfunction.

Guidelines for the assessment of RV size and function suggest the evaluation of the following parameters: end-diastolic area (EDA), end-systolic area (ESA), fractional area change (FAC), tricuspid regurgitant velocity (TRV), tricuspid annular plane systolic excursion (TAPSE), S′, RV systolic pressure (RVSP), and RVFWS.^7,8^ Some of these parameters have shown prognostic value for conditions such as pulmonary hypertension and heart failure with reduced EF.^9–11^

To date, some researchers have shown that RV parameters of size and function are variably affected during AC treatment. A meta-analysis including 1520 patients from 25 studies (73% women, mean age of 51 years) demonstrated that cancer therapy–related cardiac dysfunction is associated with worsening of right ventricular function, including a decreased fractional area change, global RV longitudinal strain, and free-wall strain, along with lower tricuspid annular plane systolic excursion and higher pulmonary artery systolic pressure; importantly, these study included a broad range of chemotherapeutic agents, not limited to AC as well as different types of malignancies such as lung and breast cancer, osteosarcoma, and lymphoma.^12^ Boczar *et al*.^13^ studied 49 patients with breast cancer who received AC and reported worsening FAC and RVFWS. However, other studies have not observed differences in RV function after chemotherapy.^14,15^ Therefore, although previous studies have evaluated right-sided heart function of patients with different cancer types undergoing AC-based chemotherapy, study sizes have been small and the data have been inconsistent.

Because the extent to which AC can compromise RV size and function is not entirely known, traditional parameters could be complemented with tools to detect subclinical RV dysfunction.^16^ RV 2-dimensional speckle tracking echocardiography, which has been included in the guidelines as an RV functional parameter, can provide additional information and potentially detect subclinical myocardial dysfunction for patients treated with AC.^6,17,18^

The primary aim of this study was to evaluate patients with lymphoma who received doxorubicin treatment and determine whether they had changes in RV size and function parameters (EDA, ESA, FAC, S′, TAPSE, RVSP, and RVFWS), derived from echocardiography at three different timepoints.

## Methods

This single-centre study was approved by the Mayo Clinic Institutional Review Board. All participants provided written, informed consent at the time of lymphoma diagnosis for use of their data for research purposes. The reporting of this study is in compliance with the STROBE (Strengthening the Reporting of Observational Studies in Epidemiology) statement.^19^

### Patient selection and data collection

We prospectively enrolled patients with Hodgkin or non-Hodgkin lymphoma treated at Mayo Clinic (Rochester, Minnesota) between 20 March 2013, and 21 February 2020. The study size was determined by performing a power calculation before study initiation; a total of 105 patients were required to detect a difference of ±3% in RVFWS, 0.03 m/s in S’, and 4 mm in TAPSE, with 95% power. Parameters of RV size and function, as referenced in the American Society of Echocardiography guidelines for the echocardiographic assessment of the right heart in adults, as well as the recommendations for cardiac chamber quantification by echocardiography in adults from the American Society of Echocardiography and the European Association of Cardiovascular Imaging, were utilized as reference values.^7,8^

All patients received a chemotherapeutic regimen that included doxorubicin (up to 350 mg/m^2^). Patients received either ABVD (doxorubicin, bleomycin, vinblastine, and dacarbazine) or R-CHOP (rituximab, cyclophosphamide, doxorubicin, vincristine, and prednisolone) regimen. Patients had an echocardiographic assessment at 3 time points: before starting treatment [T0 (baseline)], 3 to 6 (mean [SD], 5.1 [1.2]) months after starting chemotherapy (T1), and 6 to 18 (mean [SD], 12.98 [2.6]) months after starting chemotherapy (T2).

A subanalysis according to cumulative AC exposure (<200, 200–300, and >300 mg/m^2^) was performed to evaluate dose related risk.^20^

Participant medical records were reviewed at the time of enrolment. Patients were excluded if they were younger than 18 years, had previously undergone chemotherapy or radiotherapy, had a LV ejection fraction (EF) < 50% prior to chemotherapy, had more than mild valvular heart disease (insufficiency or stenosis), had a previous history of congenital heart disease or left ventricular dysfunction, or had a poor acoustic window.

### Echocardiographic assessment and image acquisition

Echocardiographic assessment was performed by a registered diagnostic cardiac sonographer following the American Society of Echocardiography and the European Association of Cardiovascular Imaging guidelines for quantifying cardiac chambers in adults.^8^ A Vivid E95 (GE HealthCare, Chicago, Illinois) with an M4S transducer was used; all measurements were performed following standardized acquisition protocols.

**Figure 1:**
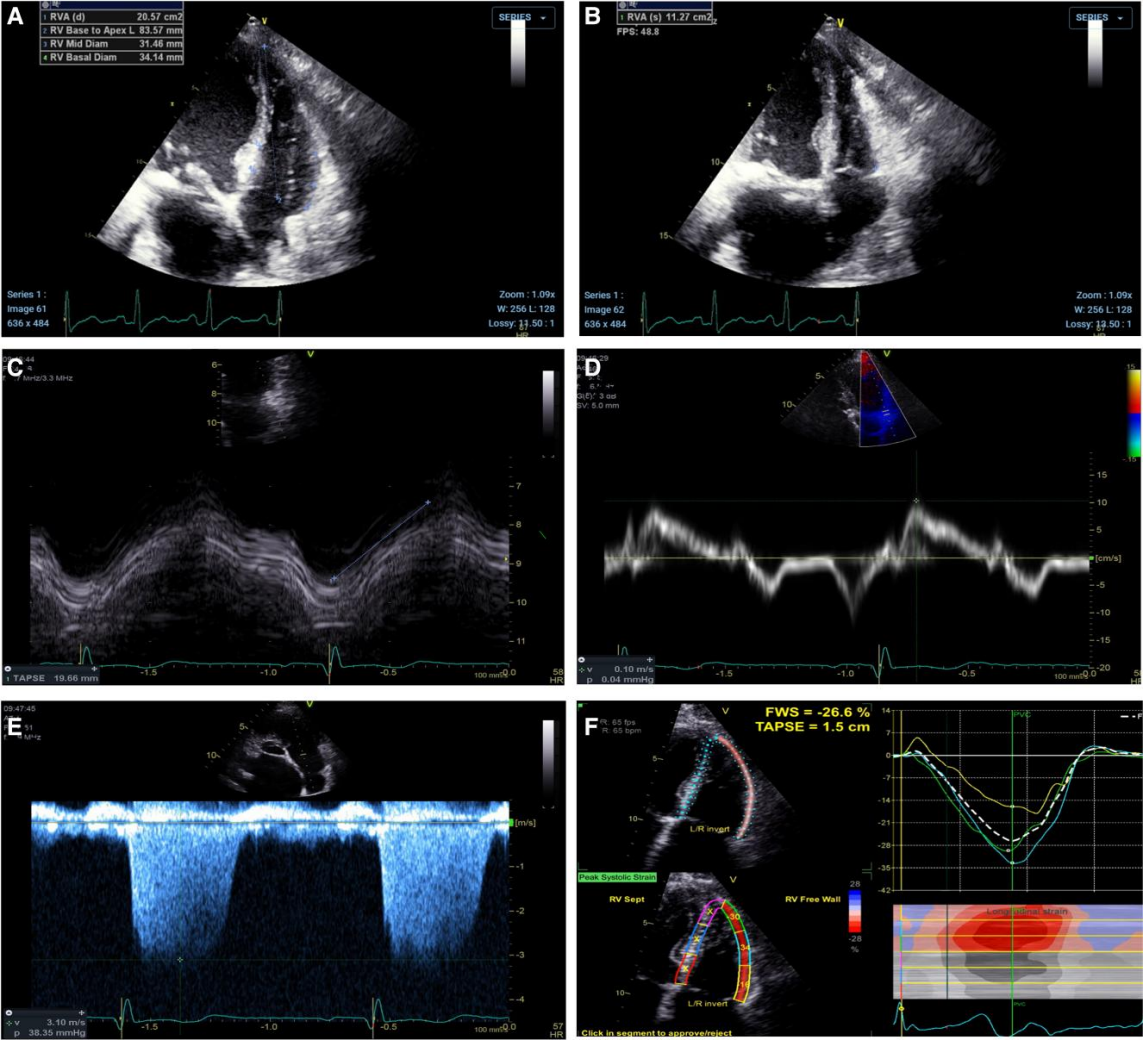
Representative echocardiographic images of the acquisition of right ventricular size and function parameters. A, end diastolic area; B, end systolic area; C, tricuspid annulus plane systolic excursion; D, S’; E, tricuspid regurgitation velocity; F, right ventricle free wall strain.

In accordance with current guidelines, the imaging protocol included a dedicated apical 4-chamber view from which EDA (*Figure 1A*) and ESA (*Figure 1B*) were obtained. FAC was calculated as follows:


FAC=(EDA−ESA)/EDA×100.

TAPSE (*Figure 1C*) was calculated in the apical 4-chamber view with M-mode: The cursor was placed through the tricuspid annulus, close to the lateral wall, with careful consideration that the cursor was parallel to the wall movement and with an angle of less than 10°. S′ (*Figure 1D*) was obtained by tissue Doppler imaging, using a pulsed Doppler cursor placed on the tricuspid annulus with optimal image alignment in this view. Continuous-wave Doppler was used to measure TRV (*Figure 1E*), capturing the highest velocity from multiple acoustic windows. This value was then used to calculate the RVSP by applying the modified Bernoulli equation and adding an estimated right atrial pressure based on inferior vena cava dimensions and collapsibility. RVFWS analysis (*Figure 1F*) was also performed during the echocardiographic assessment using the EchoPAC software in the ultrasound machine (GE HealthCare, Chicago, Illinois). Standard greyscale images of the RV were obtained from the most favourable RV-focused apical 4-chamber view, with frame rates of 40 to 90 frames/second. The RV endocardial boundaries (outlining the region of interest) were manually traced at end-systole and end-diastole to measure longitudinal strain, assuring that at least 85% of the endocardial thickness was properly tracked during the whole cardiac cycle. RVFWS was calculated as the peak strain value from the averaged strain curve derived from the three segments of the RV free wall. In 16% of patients, images that were not analysed at the time of acquisition were assessed offline with the TomTec Cardiac Performance Analysis software (TOMTEC Imaging Systems GmbH, Unterschleissheim, Germany).

Interobserver variability for RVFWS was evaluated using the mean relative error, calculated as the absolute difference between two measurements divided by the first measured value. Measurements were obtained by a second observer (E.B.M.), using offline strain analysis, for 40 previously analysed patients. We observed a strong correlation between interobserver echocardiographic measurements (mean relative error, 0.1; Pearson correlation coefficient, 0.916).

### Statistical analysis

Categorical variables were expressed as frequency and percentage, and continuous variables were reported as mean and standard deviation. The Shapiro–Wilk test was used to verify the normality of the distribution of the variables.

We stratified patients into three groups by the cumulative dosage received: less than 200 mg/m^2^, 200 to 300 mg/m^2^, and greater than 300 mg/m^2^. We used analysis of variance in conjunction with Tukey–Kramer analysis to evaluate differences between dosage groups at different time points. Missing data were addressed by including patients with complete or partial follow-up; patients with a single echocardiographic assessment were excluded. One sample *t*-tests were used to compare the obtained parameters with guideline-defined cutoff values. A *P* < 0.05 was considered statistically significant. BlueSky Statistics software (version 10.3; BlueSky Statistics, LLC) was used for all analyses.

## Results

### Baseline characteristics

Our study included 356 patients; of these, 226 (63.5%) were men, the mean (SD) age was 59.2 (16.4) years, and the body mass index (BMI) was 28.9 (5.4). 347 (97.5%) were White, 4 (1.1%) were Black or African American, 4 (1.1%) were Asian, and 1 (0.3%) was Hispanic. Cardiovascular risk factors and clinical characteristics are summarized in *Table 1*. In this cohort, 294 patients (82.6%) were diagnosed with non-Hodgkin lymphoma and 62 (17.4%) with Hodgkin lymphoma. The mean cumulative dose was 498 (173.4) mg or 243.6 (79.6) mg/m^2^ when adjusted for body surface area, and the mean number of cycles was 5.12 (1.48). The number of patients in each dosage group is shown in *Table 2*. The distribution of patients among the three doxorubicin dosage groups was similar for patients with non-Hodgkin lymphoma vs. patients with Hodgkin lymphoma (*P* = 0.75).

**Table 1:** Demographic and clinical characteristics of study participants

Variable	All participants (*n* = 356)
Age, mean (SD), y	59.2 (16.4)
Sex, no. (%)	
Male	226 (63.5)
Female	130 (36.5)
Race/ethnicity, no. (%)	
Asian	4 (1.1)
Black or African American	4 (1.1)
Hispanic	1 (0.3)
White	347 (97.5)
Clinical characteristics, mean (SD)	
Body mass index^a^	28.9 (5.4)
Systolic blood pressure, mm Hg	125 (16)
Diastolic blood pressure, mm Hg	72 (11)
Cardiovascular risk factor, no. (%)	
Hypertension	152 (42.7)
Hypertension treatment	140 (39.3)
Current smoker	19 (5.3)
Former smoker	95 (26.7)
Type 2 diabetes	47 (13.2)
Dyslipidaemia	131 (36.8)
Oncologic characteristic, no. (%)	
Hodgkin lymphoma	62 (17.4)
Non-Hodgkin lymphoma	294 (82.6)

^a^Body mass index was calculated as weight in kilograms divided by height in metres squared.

**Table 2:** Chemotherapy regimen characteristics (***n* = 356)**

Variable	Value
Cumulative anthracycline dose, mean (SD), mg	498.1 (173.4)
Cumulative BSA-adjusted anthracycline dose, mean (SD), mg/m^2^	243.6 (79.6)
Chemotherapy cycles, mean (SD), no.	5.12 (1.48)
Dose group (cumulative), no. (%)	
<200 mg/m^2^	85 (23.9)
200–300 mg/m^2^	162 (45.5)
>300 mg/m^2^	109 (30.6)

Abbreviation: BSA, body surface area.

### Echocardiographic assessment

#### General analysis (all dosages)

**Figure 2:**
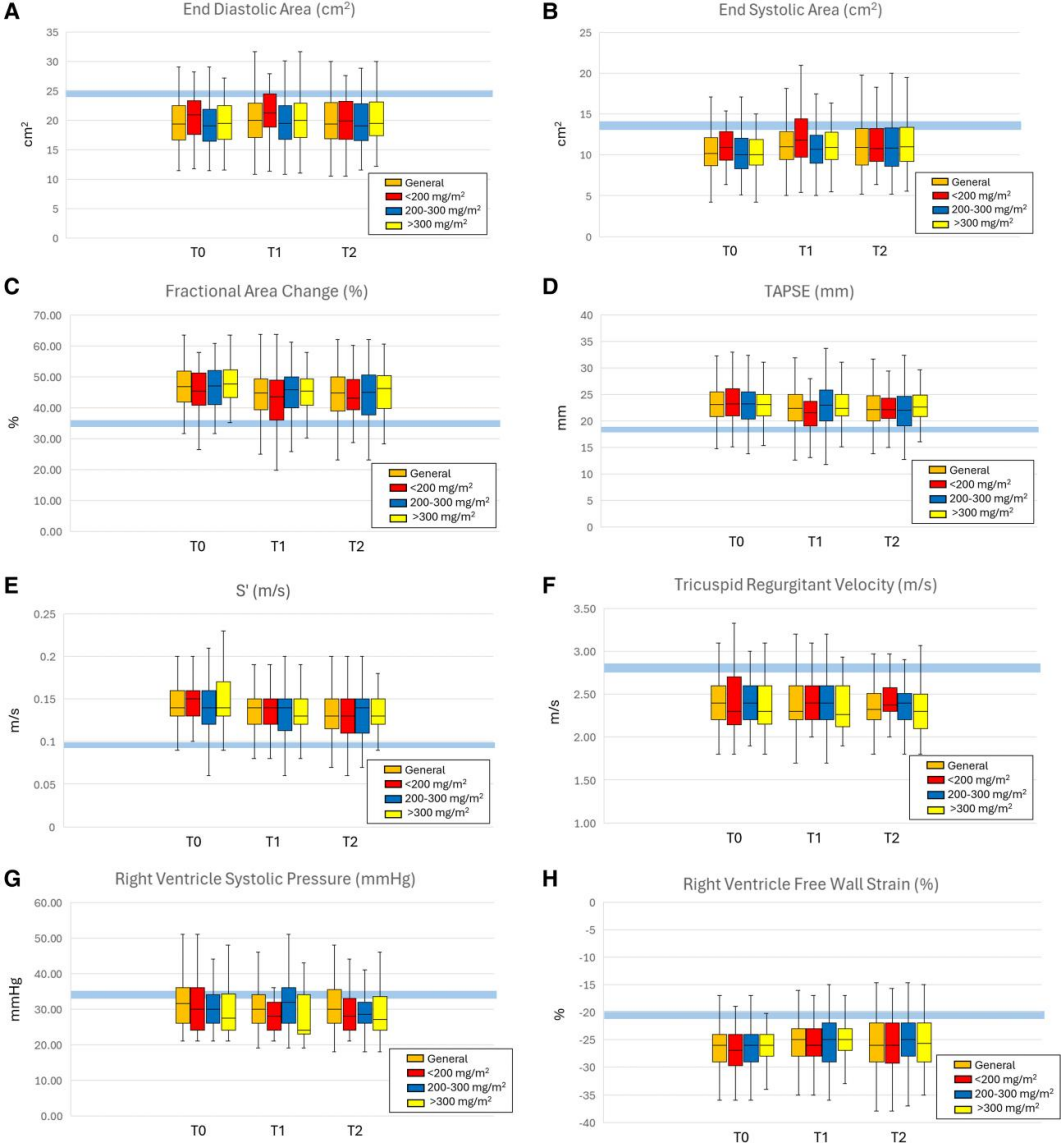
Echocardiographic measurements, general analysis and all dosages. Values were obtained at baseline (T0), 3–6 months after starting chemotherapy (T1), and 6–18 months after starting chemotherapy (T2). Light blue line shows guideline-defined reference values for each variable. A, end-diastolic area; B, end-systolic area; C, fractional area change; D, tricuspid annular plane systolic excursion (TAPSE); E, S’; F, tricuspid regurgitant velocity; G, right ventricle systolic pressure; H, right ventricle free wall strain. Reference normal values: end-diastolic area <25 cm2 , end-systolic area <14 cm^2^, FAC >35%, TRV <2.8 m/s, TAPSE >17 mm, S′ > 9.5c m/s, RVSP ≤34 mmHg, RVFWS < −20%.

Echocardiographic assessments occurred at three time points: T0, baseline; T1, 3 to 6 months after starting doxorubicin (mean [SD], 5.1 [1.2] months); and T2, 6 to 18 months (mean [SD], 12.98 [2.6] months) after starting treatment. In the general analysis, we did not identify significant differences in EDA (*Figure 2A*) measurements at T0, T1, or T2, although we observed a slight increase at T1 followed by a slight decrease at T2 (*P* = 0.12). ESA (*Figure 2B*) showed slight changes over time, with an increase at T1 and stabilization at T2 (*P* < 0.001). FAC (*Figure 2C*) decreased from T0 to T1 and remained stable at T2 (*P* = 0.003). TAPSE (*Figure 2D*) values showed small reductions across time points, decreasing at T1 and further at T2 (*P* = 0.006). Similarly, S′ (*Figure 2E*) decreased from T0 to T1 and remained unchanged at T2 (*P* < 0.001). No changes over time were observed for TRV (*P* = 0.58) (*Figure 2F*) and RVSP (*P* = 0.75) (*Figure 2G*). In contrast, RVFWS (*Figure 2H*) values showed a minimal decrease from T0 to T1, with little further change at T2 (*P* = 0.001). Values remained within guideline parameters all time points (*P* < 0.001) (*Figure 2* and *Table 3*).

**Table 3:** Echocardiographic measurements, general analysis (all dosages), stratified by time point^a^

Variable	All participants (*n* = 356)				Guideline defined normal values*
	T0	T1	T2	*P* value	
End-diastolic area, mean (SD), cm^2^	19.68 (4.15)	20.35 (4.39)	20.18 (4.64)	0.12	<25
End-systolic area, mean (SD), cm^2^	10.55 (2.85)^b^	11.41 (3.36)	11.32 (3.50)	<0.001	<14
FAC, mean (SD), %	46.67 (6.74)^b^	44.35 (7.82)	44.40 (7.75)	0.003	>35
TRV, mean (SD), m/s	2.41 (0.36)	2.40 (0.31)	2.38 (0.29)	0.58	<2.8
TAPSE, mean (SD), mm	23.17 (4.14)^b^	22.39 (4.10)	22.13 (3.88)	0.006	>17
S′, mean (SD), m/s	0.14 (0.03)	0.13 (0.02)	0.13 (0.02)	<0.001	0.095
RVSP, mean (SD), mm Hg	30.57 (7.00)	29.82 (6.93)	29.48 (6.63)	0.75	34
RVFWS, mean (SD), %^c^	−26.29 (3.99)^b^	−25.15 (4.48)	−25.37 (4.83)	0.001	−20

Abbreviations: FAC, fractional area change; RVFWS, right ventricular free wall strain; RVSP, right ventricular systolic pressure; TAPSE, tricuspid annulus plane systolic excursion; TRV, tricuspid regurgitant velocity.

^*^All values remained within guideline-defined normal values at all time points (*P* < 0.001).

^a^Values were obtained at baseline (T0), 3 to 6 months after starting chemotherapy (T1), and 6 to 18 months after starting chemotherapy (T2).

^b^Statistically significant difference between T0 and T1 and between T0 and T2.

^c^RVFWS was assessed at each visit for 300 patients (84%); 56 patients (16%) had offline strain analysis if RVFWS was not initially evaluated.

#### Doxorubicin dosage less than 200 mg/m^2^

Among the patients who received less than 200 mg/m^2^ of doxorubicin (*Figure 2* and *Table 4*), EDA (*Figure 2A*) remained stable across T0, T1, and T2, with a slight increase at T1, followed by a return to baseline at T2 (*P* = 0.35). ESA (*Figure 2B*) values increased from T0 to T1, followed by a decrease at T2 (*P* = 0.02). Similarly, FAC (*Figure 2C*) values decreased at T1 and showed partial recovery at T2 (*P* = 0.03). TAPSE (*Figure 2D*) values decreased from T0 to T1, with a slight increase at T2 (*P* < 0.001). S′ (*Figure 2E*) also decreased from T0 to T1 and remained stable at T2 (*P* = 0.01). Neither TRV nor RVSP showed changes over time (*Figure 2F* and *G*). In contrast, RVFWS (*Figure 2H*) remained stable (no differences observed) across all three time points (*P* = 0.18). Values remained within guideline-defined normality thresholds at every assessed time point (*P* < 0.001).

**Table 4:** Echocardiographic measurements of patients who received less than 200 mg/m^2^ doxorubicin, stratified by time point^a^

Variable	T0	T1	T2	*P* value	Guideline defined normal values*
End-diastolic area, mean (SD), cm^2^	20.42 (3.66)	21.28 (3.89)	20.46 (4.78)	0.35	<25
End-systolic area, mean (SD), cm^2^	11.15 (2.46)^b^	12.49 (3.73)	11.48 (3.40)	0.02	<14
FAC, mean (SD), %	45.37 (6.74)^b^	42.16 (9.29)	44.13 (7.60)	0.03	>35
TRV, mean (SD), m/s	2.42 (0.54)	2.43 (0.32)	2.45 (0.35)	0.91	<2.8
TAPSE, mean (SD), mm	23.91 (4.02)^b,c^	21.14 (4.28)	21.98 (3.78)	<0.001	>17
S′, mean (SD), m/s	0.15 (0.03)^b,c^	0.13 (0.03)	0.13 (0.03)	0.01	0.095
RVSP, mean (SD), mm Hg	31.55 (7.95)	28.00 (4.62)	29.37 (6.53)	0.10	34
RVFWS, mean (SD), %	−26.61 (3.95)	−25.27 (4.86)	−26.07 (4.94)	0.18	−20

Abbreviations: FAC, fractional area change; RVFWS, right ventricular free wall strain; RVSP, right ventricular systolic pressure; TAPSE, tricuspid annulus plane systolic excursion; TRV, tricuspid regurgitant velocity.

^*^All values remained within guideline-defined normal values at all time points (*P* < 0.001).

^a^Values were obtained at baseline (T0), 3–6 months after starting chemotherapy (T1), and 6–18 months after starting chemotherapy (T2).

^b^Statistically significant difference between T0 and T1.

^c^Statistically significant difference between T0 and T2.

#### Doxorubicin dosage 200–300 mg/m^2^

Among the patients who received 200–300 mg/m^2^ (*Figure 2* and *Table 5*), EDA (*Figure 2A*) showed almost no change across time points, with values remaining stable from T0 to T2 (*P* = 0.32). ESA (*Figure 2B*) values increased mildly from T0 to T1 and continued to increase slightly at T2, but the difference was not statistically significant (*P* = 0.07). FAC (*Figure 2C*) values decreased from T0 to T1 and decreased further at T2 (*P* = 0.02). TAPSE (*Figure 2D*) values remained largely unchanged over time (*P* = 0.20). S′ (*Figure 2E*) decreased from T0 to T1 (*P* = 0.008). The remaining parameters did not show changes over time (*Figure 2F* and *G*). RVFWS (*Figure 2H*) values showed a marginal decline from T0 to T1, with a further decrease at T2 (*P* = 0.05). Values remained within guideline-defined normal thresholds at all time points evaluated (*P* < 0.001).

**Table 5:** Echocardiographic measurements of patients who received 200 to 300 mg/m^2^ doxorubicin, stratified by time point^a^

Variable	T0	T1	T2	*P* value	Guideline defined normal values*
End-diastolic area, mean (SD), cm^2^	19.41 (4.68)	20.08 (4.80)	19.98 (4.94)	0.32	<25
End-systolic area, mean (SD), cm^2^	10.46 (3.31)	11.13 (3.53)	11.29 (3.86)	0.07	<14
FAC, mean (SD), %	46.57 (7.05)^b^	45.06 (7.46)	44.29 (7.89)	0.02	>35
TRV, mean (SD), m/s	2.44 (0.29)	2.42 (0.33)	2.38 (0.27)	0.36	<2.8
TAPSE, mean (SD), mm	22.71 (4.37)	22.59 (4.17)	21.86 (4.33)	0.20	>17
S′, mean (SD), m/s	0.14 (0.03)^b,c^	0.13 (0.03)	0.13 (0.03)	0.008	0.095
RVSP, mean (SD), mm Hg	30.59 (6.56)	32.08 (7.75)	29.73 (6.15)	0.11	34
RVFWS, mean (SD), %	−26.13 (4.52)	−25.41 (4.49)	−24.90 (4.95)	0.05	−20

Abbreviations: FAC, fractional area change; RVFWS, right ventricular free wall strain; RVSP, right ventricular systolic pressure; TAPSE, tricuspid annulus plane systolic excursion; TRV, tricuspid regurgitant velocity.

^*^All values remained within guideline-defined normal values at all time points (*P* < 0.001).

^a^Values were obtained at baseline (T0), 3 to 6 months after starting chemotherapy (T1), and 6 to 18 months after starting chemotherapy (T2).

^b^Statistically significant difference between T0 and T2.

^c^Statistically significant difference between T0 and T1.

#### Doxorubicin dosage greater than 300 mg/m^2^

Among the patients who received greater than 300 mg/m^2^ of doxorubicin (*Figure 2* and *Table 6*), EDA (*Figure 2A*) showed almost no change over time, remaining stable from T0 through T2 (*P* = 0.36). ESA (*Figure 2B*) values increased progressively from T0 to T1 and continued to increase at T2, indicating greater RV size over time (*P* = 0.01). FAC (*Figure 2C*) values showed a decrease from T0 to T1, with further reduction at T2 (*P* = 0.002). TAPSE (*Figure 2D*) decreased only slightly over time (*P* = 0.46). S′ (*Figure 2E*) decreased from T0 to T1 and remained reduced at T2 (*P* = 0.002). TRV and RVSP remained stable throughout the follow up (*Figure 2F* and *G*). RVFWS (*Figure 2H*) also showed a decrease from T0 to T1, with partial recovery at T2 (*P* = 0.01). Values consistently remained in the guideline-defined normal parameters at all time points (*P* < 0.001).

**Table 6:** Echocardiographic measurements of patients who received greater than 300 mg/m^2^ doxorubicin, stratified by time point^a^

Variable	T0	T1	T2	*P* value	Guideline defined normal values*
End-diastolic area, mean (SD), cm^2^	19.53 (3.60)	20.07 (4.06)	20.32 (4.12)	0.36	<25
End-systolic area, mean (SD), cm^2^	10.21 (2.31)^b,c^	11.06 (2.63)	11.28 (3.01)	0.01	<14
FAC, mean (SD), %	47.84 (6.13)^b,c^	44.92 (6.93)	44.78 (7.81)	0.002	>35
TRV, mean (SD), m/s	2.38 (0.30)	2.34 (0.28)	2.34 (0.30)	0.76	<2.8
TAPSE, mean (SD), mm	23.33 (3.81)	22.92 (3.75)	22.66 (3.16)	0.46	>17
S′, mean (SD), m/s	0.15 (0.03)^b,c^	0.13 (0.02)	0.14 (0.02)	0.002	0.095
RVSP, mean (SD), mm Hg	29.74 (7.09)	27.82 (6.17)	29.20 (7.53)	0.46	34
RVFWS, mean (SD), %	−26.30 (3.12)^b^	−24.71 (4.18)	−25.61 (4.55)	0.01	−20

Abbreviations: FAC, fractional area change; RVFWS, right ventricular free wall strain; RVSP, right ventricular systolic pressure; TAPSE, tricuspid annulus plane systolic excursion; TRV, tricuspid regurgitant velocity.

^*^All values remained within guideline-defined normal values at all time points (*P* < 0.001)

^a^Values were obtained at baseline (T0), 3–6 months after starting chemotherapy (T1), and 6–18 months after starting chemotherapy (T2).

^b^Statistically significant difference between T0 and T1.

^c^Statistically significant difference between T0 and T2.

## Discussion

To the best of our knowledge, this is the first study to comprehensively evaluate right-ventricular volumetric and functional parameters in a large cohort of patients with lymphoma treated with AC, including a dedicated subanalysis by cumulative doses. Our main finding was that these parameters remained within the guideline reference values defined by echocardiography to a follow-up of 12.98 ± 2.6 months.

Tanindi *et al*.^21^ reported that RV functional parameters decreased specifically during chemotherapy for 37 patients with a recent breast cancer diagnosis who received six cycles of doxorubicin (60 mg/m^2^). That cohort showed a reduction in FAC from 63.7% (3.6%) at baseline to 63.3% (3.7%) after the first cycle and then to 61.2% (4.4%) after the second cycle. TAPSE also decreased from 18.2 (2.0) mm (baseline) to 17.8 (1.9) mm (after cycle 1) and to 16.2 (2.4) mm (after cycle 2). However, when comparing values measured before and after AC initiation, they generally remained within guideline-defined normal values. Similar changes were seen in our population, particularly at T1, with both FAC and TAPSE decreasing slightly. Although our measured values were not as high as those reported previously (FAC of 63.3% and 61.2% vs. 44.35% and 44.40% in the current study), our greater sample size enabled a more comprehensive analysis that enhanced statistical power and ensured that our findings were more representative of the broader population. Our longitudinal design with three time points strengthened the study findings by allowing evaluation of temporal trends and a comprehensive assessment of changes over time and different AC doses.

Anqi *et al*.^14^ focused on sequential assessments (baseline and after the second, fourth, and sixth chemotherapy cycles) for 40 patients with breast cancer [AC dosage, 363.90 (107.77) mg/m^2^]. Although they observed worsening LV parameters, RV parameters, such as TAPSE, S′, RV diameters, and linear measurements remained unchanged. Their results are similar to our findings, in which all parameters evaluated remained within guideline-defined reference values. In our study, RVFWS in the general analysis (all dosages) showed a decrease from −26.29% (3.99%) to −25.15% (4.48%) between T0 and T1. Curiously, in the AC dosage subgroup analysis, only the highest-dosage (>300 mg/m^2^) showed a difference in the RVFWS when T0 [−26.30% (3.12%)], T1 [−24.71% (4.18%)], and T2 [−25.61% (4.55%)] were compared (*P* = 0.01). However, the change in RVFWS values for the 200 to 300 mg/m^2^ group was not statistically significant (*P* = 0.05).

Chang *et al*.^22^ emphasized the role of RVFWS as a key marker of RV dysfunction for 35 patients with breast cancer who planned to receive 6–8 cycles of epirubicin [354.19 (336.08) mg/m^2^]. Their study showed RVFWS decreasing with treatment (baseline, −22.4% [4.97%]; after cycle 1, −18.48% [4.46%]; after cycle 3, −16.86% [7.27%]). The study reported that accumulating doses of epirubicin positively correlated with the decline in RVFWS and dyspnoea development and that a lower RVFWS predicted the development of dyspnoea. TAPSE also decreased with time. Although the parameters previously mentioned were slightly affected in our study, we did not observe a dose-dependent effect with doxorubicin when these RV parameters were assessed. These discrepancies could be due to the different types of chemotherapeutic agents used, as well as the type of malignancy assessed. Compared with other studies, our study included a larger sample size and a prospective design with a follow up of 12.98 ± 2.6 months.

Abdar Esfahani *et al*.^23^ showed changes in RV function after 6 months of chemotherapy in 49 patients with breast cancer receiving 450–550 mg/m^2^ of AC. Specifically, they noted a marked increase in RV end-diastolic diameter, a decrease in FAC (from 49.83% to 43.59%) and TAPSE (from 18.8 to 17.7 mm), and an increase in systolic pulmonary arterial pressure (from 20.63 to 22.24 mm Hg). These findings align with our results, which also showed minimal decreases in FAC and TAPSE over time (although we saw no changes in RVSP), indicating a slight decrease in RV systolic function during chemotherapy. Similarly, all assessed parameters remained within guideline-defined normal values throughout the study.

Wang *et al*.^24^ evaluated RV function after AC treatment [cumulative dosage, 311.2 (99.8) mg/m^2^] for 61 patients with diffuse large B-cell lymphoma. They echocardiographically assessed patients at baseline, after the third cycle, after therapy completion, and 10 months after chemotherapy initiation. They reported a decrease in TAPSE (from 22.5 [3.2] mm at baseline to 19.8 [2.6] mm at follow-up), S′ (0.13 [1.0] to 0.11 [1.0] m/s), and RVFWS (−25.8% [3.8%] to −23.2% [3.4%]) and an increase in end-systolic and end-diastolic volumes. Our study identified a similar trend in these parameters, and like the aforementioned study, no statistically significant differences were seen regarding AC dosage received and echocardiographic parameters worsening. Both studies’ results remained largely within guideline-defined reference values; by incorporating a larger cohort, assessing more variables, and ensuring a prolonged follow-up, our study enhanced the credibility and validity of its conclusions.

Our study offers insight into the effects of AC chemotherapy on RV function for patients with lymphoma. We addressed limitations of previous research by incorporating eight echocardiographic parameters, leading to more comprehensive and reliable results on changes in RV function and structure. The methodology used, including detailed echocardiographic assessments across multiple time points and dosage groups, as well as the prospective data collection, enabled an in-depth understanding of RV alterations over time.

### Limitations

The findings of this single-centre study may lack generalizability to other patient populations. The sexes were not equally represented in our study, and most patients were older than 50 years (the age range most affected by non-Hodgkin lymphoma). The study cohort was predominantly White. RV strain was assessed offline for 16% of patients, which may have introduced variability in the measurements, but a strong correlation between interobserver echocardiographic measurements was found (Pearson correlation coefficient = 0.916). Furthermore, RV EF data were not obtained because the RV’s complex geometry requires advanced echocardiographic techniques that were not available during the study period.

### Conclusions

To the best of our knowledge, our work describes the largest, single-centre study evaluating the effect of different AC dosages on parameters of RV size and function derived by echocardiography. These findings suggest that, in this population, doxorubicin with cumulative doses of up to 350 mg/m^2^ does not have an early effect on RV volumetric and functional parameters when compared with guideline-derived values. Future studies evaluating the influence of cardiovascular risk factors in this patient population would provide valuable information.

## Lead author biography


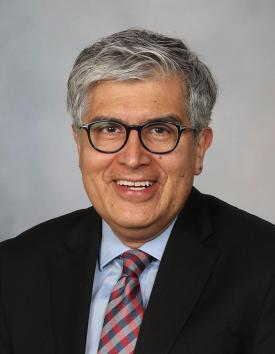


Dr. Hector R. Villarraga is a cardiologist at the Mayo Clinic in Rochester, Minnesota.

He is an Associate Professor of Medicine at the Mayo Clinic College of Medicine. He is board certified in Cardiovascular Diseases, Internal Medicine, and Echocardiography. His research interests include evaluation of myocardial mechanical function by speckle tracking echocardiography (strain) in cardiomyopathies with normal ejection fraction and in cardio-oncology.

He is a member of the Cardio-Oncology Section Leadership Council of the InterAmerican Society of Cardiology (SIAC) and former member of the Cardio-Oncology section leadership council of the American College of Cardiology (ACC).

Current prospective trials include evaluation of early cardiotoxicity in patients with lymphoma, breast cancer, sarcoma, and patients undergoing proton and photon radiotherapy as well as cardiovascular risk factors involved in the development of MACE. He has over 100 published manuscripts and book chapters.

## Data Availability

The data set used in this study contains patient information and is therefore not publicly available. Access to the data is restricted to researchers within Mayo Clinic, in accordance with institutional and ethical guidelines. Requests for access may be considered on a case-by-case basis and require approval from the relevant institutional review board or ethics committee.
